# Associations of Social Deprivation and Oncology Physician Network Vulnerability With Acute Care Utilization in the SEER‐Medicare Population

**DOI:** 10.1111/1475-6773.70070

**Published:** 2025-11-18

**Authors:** Ashlee A. Korsberg, Gabriel A. Brooks, A. James O'Malley, Tracy Onega, Andrew P. Schaefer, Erika L. Moen

**Affiliations:** ^1^ Department of Biomedical Data Science Dartmouth Geisel School of Medicine Lebanon New Hampshire USA; ^2^ Department of Medicine Dartmouth Hitchcock Medical Center Lebanon New Hampshire USA; ^3^ The Dartmouth Institute for Health Policy and Clinical Practice Lebanon New Hampshire USA; ^4^ Dartmouth Cancer Center, Dartmouth Hitchcock Medical Center Lebanon New Hampshire USA; ^5^ Department of Population Health Sciences and Huntsman Cancer Institute University of Utah Salt Lake City Utah USA

**Keywords:** acute care utilization, cancer care delivery, patient‐sharing networks, physician network vulnerability, SEER‐Medicare, social deprivation index

## Abstract

**Objective:**

The objectives of this study were to evaluate associations of social deprivation with acute care utilization among patients with cancer, and to examine potential effect modification by physician network vulnerability.

**Study Setting and Design:**

For this retrospective cohort study, the primary exposure variable was neighborhood‐level socioeconomic disadvantage, operationalized through the social deprivation index (SDI). We assembled physician patient‐sharing networks and calculated a measure of network vulnerability for each referral region to capture specialist scarcity. The two outcomes of interest were counts of emergency department (ED) visits and non‐elective hospitalizations during the 12 months following cancer diagnosis. We conducted hurdle regressions, with logistic and negative binomial mixed‐effects models for the zero and positive, non‐zero parts of the outcome distribution, respectively, and stratified by physician network vulnerability.

**Data Sources and Analytic Sample:**

We analyzed 2016–2020 Surveillance, Epidemiology, and End Results (SEER)‐Medicare linked data for Medicare beneficiaries diagnosed with breast, colorectal, or lung cancer.

**Principal Findings:**

The study cohort comprised 47,756 patients with breast, colorectal or lung cancer. Patients in high SDI neighborhoods (vs. low) had a higher probability of at least one ED visit across all physician network vulnerability strata (low network vulnerability—average marginal effect (AME) [95% CI]: 0.03 [0.01–0.05]; medium network vulnerability—AME [95% CI]: 0.03 [0.01–0.04]; high network vulnerability—AME [95% CI]: 0.05 [0.02–0.08]). Conditional on at least one ED visit, patients in high SDI neighborhoods (vs. low) had a greater relative risk of additional ED visits when their region was characterized by low physician network vulnerability (RR [95% CI]: 1.25 [1.09–1.43]).

**Conclusions:**

Our findings suggest that SDI and physician network vulnerability interact to increase the probability and likelihood of ED visits, but the interaction was minimal for non‐elective hospitalizations. More research is needed to better understand how social drivers of health and oncology workforce scarcity affect care utilization and outcomes in patients with cancer.

**Summary**

- What is known on this topic
  ○ Neighborhood‐level socioeconomic disadvantage is a critical upstream social driver of health, and there is growing evidence that greater social deprivation is associated with worse outcomes among patients with cancer.
  ○ Higher physician network vulnerability, a measure derived from patient‐sharing networks that captures regional specialist scarcity, has been associated with lower access to high quality care among patients with cancer.
- What this study adds
  ○ The interplay between social deprivation and oncology physician workforce scarcity in care utilization during cancer treatment has been understudied, but may be substantial.
  ○ This study provides a novel examination of how neighborhood SDI is associated with acute care utilization in different oncology scarcity settings among patients with cancer.
  ○ Our findings suggest that multi‐level vulnerabilities may have more of an influential effect on emergency department utilization than non‐elective hospitalizations among patients with cancer.

## 1 Introduction

Neighborhood‐level socioeconomic disadvantage is a critical upstream social driver of health (SDOH) that can impact health outcomes. Social deprivation index (SDI) is used in health services research to study associations between neighborhood socioeconomic deprivation and health outcomes [1, 2]. There is growing evidence that greater SDI is associated with worse outcomes among patients with cancer, such as lower colorectal cancer screening rates, more advanced stage at diagnosis for gastrointestinal and breast cancer, lower likelihood of receiving guideline‐concordant care for colorectal cancer, and increased mortality for liver, lung, colorectal, pancreatic and pediatric cancers [3, 4, 5, 6, 7, 8, 9, 10, 11, 12, 13]. Although the majority of the evidence shows a significant relationship, some studies have reported that SDI is not associated with outcomes among patients with cancer, including advanced stage at diagnosis for lung or colorectal cancer, rates of colorectal cancer screening, odds of surgical treatment for colorectal, lung, pancreatic, breast or laryngeal cancers, worse end‐of‐life cancer care, or survival rates for pancreatic cancer [7, 13, 14, 15, 16, 17, 18]. Given that SDI takes into account area‐level poverty, education, unemployment, and access to care rates, it is commonly used as a proxy for individual‐level financial, physical, and social challenges. It is hypothesized that patients with cancer experiencing these challenges face greater barriers to accessing and adhering to the complexities of cancer care delivery, which may lead them to delay care until absolutely necessary; consequently, we hypothesize they may have a higher risk of non‐elective hospitalizations and emergency department (ED) visits during cancer treatment.

A compounding factor to worse health outcomes among individuals residing in more socially deprived neighborhoods could be lower access to specialized cancer services. After cancer diagnosis, patients receive cancer care from complex networks of physicians. A robust oncology network is essential for delivering cancer care that is well‐coordinated, high quality, and timely. The literature shows that strong multi‐disciplinary teams result in better patient outcomes and lower care costs [19, 20]. Few studies have considered the interplay between access to multidisciplinary cancer specialists and neighborhood deprivation in determining cancer health outcomes.

Network analysis is increasingly being used to study how the organization of relationships between physicians relates to health care utilization and patient outcomes [21, 22, 23]. Medical claims can be used to create networks of physicians based on the extent to which they share patients [24]. These “patient‐sharing” networks have been shown to reflect self‐reported referral and advice seeking relationships among physicians [25]. Studies have found that higher network density, a measure of physician connectedness, is associated with lower odds of hospitalizations and readmissions, and lower healthcare costs [26, 27]. Prior work has developed a measure termed physician network vulnerability to capture regions with oncology specialist scarcity using a physician linchpin score. Higher physician network vulnerability (e.g., higher proportion of linchpin physicians) has been associated with lower access to multidisciplinary cancer consultations and lower likelihood of receiving radiation therapy [28].

**FIGURE 1:**
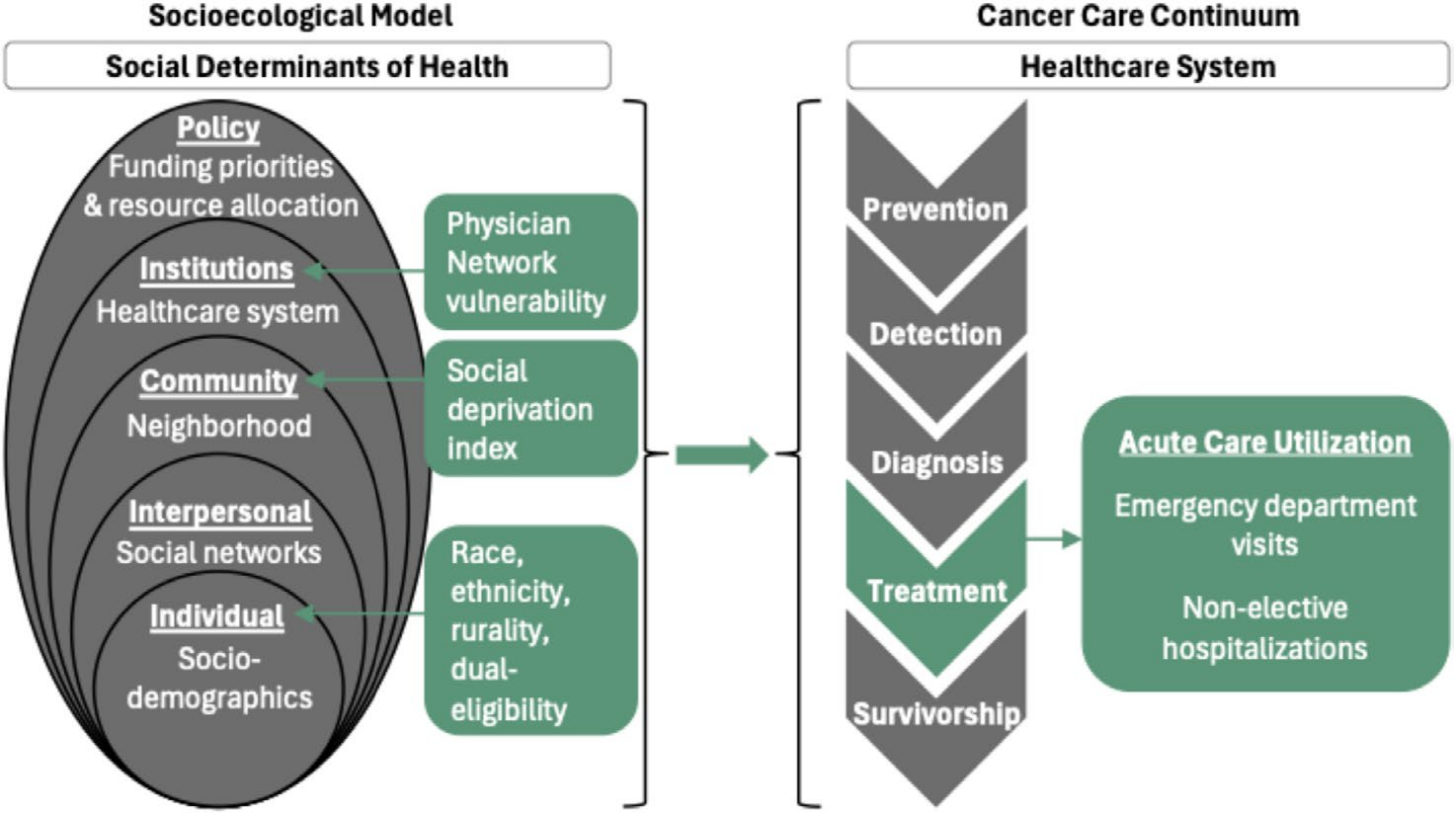
Socio‐ecological model of health and cancer care continuum framework.

Recognizing the role of multiple levels of influence on cancer health outcomes and the possible interactions among them, we used the socioecological model and cancer care continuum framework to examine acute care utilization among patients with cancer (Figure 1). In health services research, the socioecological model is used to study how drivers of health from five different levels—personal, interpersonal, community, institutional, and policy—interact to influence health status and behaviors [29, 30, 31]. The cancer care continuum describes the major stages of the cancer care journey—prevention, detection, diagnosis, treatment and survivorship—and is used to study the complex care needs of patients with cancer [32]. For this study, we used the socioecological model and the cancer care continuum to explore how individual‐, community‐ (i.e., neighborhood‐), and institutional (i.e., health system‐) level drivers of health interact to influence acute care utilization among patients with cancer. Although multilevel vulnerabilities influence outcomes at every stage of the cancer care journey, this study specifically focuses on acute care utilization during the treatment phase. Ultimately, both of these frameworks can be used to develop strategies that target multiple levels of influence to improve cancer care delivery across the care continuum.

The objectives of this study were to evaluate associations of SDI with acute care utilization among patients with cancer and to examine potential effect modification by physician network vulnerability. We used SDI to represent a neighborhood‐level driver of health and physician network vulnerability to reflect a health system‐level factor. We defined acute care utilization as ED visits and non‐elective hospitalizations. We hypothesized patients residing in high SDI neighborhoods (vs low) would have greater acute care utilization, and that the effect size would be greatest in regions characterized as high physician network vulnerability. The current literature has mainly focused on how acute care utilization differs by clinical and treatment factors. Our study aimed to build on this research by gaining insight into variation in acute care utilization across regions by studying the interaction between multilevel drivers of health.

## 2 Methods

### 2.1 Data Sources and Study Population

We used the Surveillance, Epidemiology, and End Results (SEER)‐Medicare linked database, which includes SEER registry data from the National Cancer Institute (NCI) and Medicare claims from the Centers for Medicare and Medicaid Services (CMS). SEER data come from population‐based cancer registries that cover about 48% of the US population. These data include detailed clinical information on new cancer cases such as cancer site, stage at diagnosis, and histology [33]. The Medicare claims included in this linkage and used in this study are covered services under Part A (hospital) and Part B (any type of outpatient care and physician services).

For our analytic cohort, we identified Medicare beneficiaries with a first diagnosis of breast, colorectal and lung cancer in 2018–2019 from the SEER data. We included patients aged 66 to 99 years old, and those enrolled in Medicare fee‐for‐service Parts A and B in the 12 months preceding and 12 months following their initial diagnosis with no HMO enrollment. Additionally, we only included patients with non‐missing ZIP codes located within the United States (Figure S1).

We mapped 2018–2019 census tract‐level SDI score data from the Robert Graham Center to patients' residential ZIP codes in the SEER‐Medicare data. The SDI scores range between 0 and 100, where 0 indicates least and 100 indicates most, socioeconomically disadvantaged neighborhoods. The components are the percentages of residents in a census tract living in poverty, with less than 12 years of education, in single‐parent households, living in rented housing units, living in overcrowded housing units, households without a car, and non‐employed adults < 65 years old as measured by the American Community Survey. We used a census tract to 5‐digit ZIP code crosswalk to map census tracts in the SDI data to ZIP codes in the SEER‐Medicare data. For each ZIP, we identified all census tracts that overlapped, then multiplied each census tract's SDI score by the proportion of residents that lived in the ZIP; these census tract‐level weighted SDI scores were summed to get the ZIP code‐level SDI score. These ZIP code‐level SDI scores were linked to patients' residential ZIP codes. The ZIP code‐level SDI scores were normally distributed across patients, so we created low, medium and high SDI categories based on distribution tertiles. We used 2018 SDI data for patients with an initial cancer diagnosis in 2018 and 2019 SDI data for patients with an initial cancer diagnosis in 2019.

### 2.2 Network Assembly and Network Measures

We constructed patient‐sharing networks for each hospital referral region (HRR) using Part B claims from patients diagnosed with breast, colorectal, and lung cancer in 2016–2017. We chose to assemble the cohort using data that precedes our analytic cohort to preclude patient encounters contributing to both the effect modifier (physician network vulnerability) and the outcomes (ED visits and non‐elective hospitalizations). Physicians were connected in the network if they shared at least two patients. Each physician was assigned to a ZIP code based on the plurality of Part B claims. In instances where the number of claim encounters was the same for more than one ZIP code, the physician was assigned to the ZIP code where their volume of unique patients was highest. These ZIP code assignments were then mapped to HRRs. Physicians with no encounters in a ZIP code that mapped to a HRR were not included in the patient‐sharing networks (Figure S2).

Linchpin scores for medical, radiation and surgical oncologists were calculated from the 2016–2017 patient‐sharing network. The linchpin score represents the scarcity of a specialty type within a patient‐sharing network. For example, the linchpin score for a medical oncologist is calculated by summing their patient‐sharing ties with physicians who have no other connections to a medical oncologist and dividing by their total sum of patient‐sharing ties (Figure S3) [34]. Physician specialty was identified from the Medicare Data on Provider Practice and Specialty (MDPPS) file. For each oncology specialty (medical, radiation, and surgical), physicians were identified as linchpins if their score was in the top 15% of their specialty's linchpin score distribution.

Next, we calculated HRR physician network vulnerability based on the proportion of oncology physicians who were classified as linchpins. For each HRR, the proportion of linchpin oncology physicians was calculated as the number of linchpin oncology physicians (medical + radiation + surgical) divided by the total number of oncology physicians. Then, we visualized the distribution of the proportion of linchpin oncology physicians across HRRs, and identified thresholds to categorize each HRR as low, medium or high physician network vulnerability. HRRs categorized as low represent regions with less oncologist scarcity (i.e., lower proportion of linchpin physicians) and those categorized as high represent regions with more oncologist scarcity (i.e., higher proportion of linchpin physicians).

### 2.3 Study Variables

#### 2.3.1 Outcomes

The conceptual outcome of interest was acute care utilization during the treatment phase of care among the analytic cohort. We operationalized this as total counts of ED visits and total counts of non‐elective hospitalizations during the 12 months after an incident cancer diagnosis. We identified these outcomes from the Part B, outpatient and MedPAR 2018–2020 claims. ED visits were identified in the Part B and outpatient claims based on CPT codes 99281–99285; revenue codes 0450–0452, 0459, and 0981; and place of service code 23. Non‐elective hospitalizations were identified from MedPAR claims based on non‐elective admission type codes or an admission from the ED. These codes were selected based on ResDAC guidance and other key publications [35, 36, 37].

#### 2.3.2 Main Exposures

There were two main exposure variables: neighborhood deprivation and physician network vulnerability. Patients were assigned to a HRR based on their residential ZIP codes, and inherited the physician network vulnerability group (low/medium/high) of the HRR. Patients were assigned to a neighborhood deprivation group (low/medium/high) based on the methodology described above.

#### 2.3.3 Covariates

Patient age, sex, race, ethnicity, rurality of residence (rurality), Yost index quintile, cancer type, cancer stage, and year of diagnosis were identified from the SEER Cancer file. Race and ethnicity were included in the analyses to capture the effects of structural racism, as this plays a critical role in access to care, healthcare utilization, and health outcomes. Patient rurality was determined using the Rural Urban Continuum Code (RUCC) system; patients were categorized as residing in a metropolitan, micropolitan, or rural setting based on their residential ZIP code. The Yost index is a county‐level measure of socioeconomic status provided in the SEER file and was retained as a descriptive covariate, but was not included in analytic models due to collinearity with SDI [38]. Medicare‐Medicaid dual‐eligibility status (dual‐eligibility) was identified from the Medicare Beneficiary Summary File. Comorbidities were identified using the Klabunde method, which flags Charlson comorbidities in MedPAR, Outpatient, and Carrier claims based on diagnosis codes during the 12 months preceding initial cancer diagnosis. The oncology physician count per beneficiary by HRR was calculated from the Part B 2016–2017 claims. The Medicare Advantage penetration rate in the patient's state of residence was derived from the Center for Medicare and Medicaid's Medicare Advantage State Penetration monthly reports [39]. We calculated the average monthly Medicare Advantage penetration rate, by state, from 2016 to 2017.

### 2.4 Statistical Analyses

We used bivariate analyses to evaluate the associations of patient characteristics with physician network vulnerability region and SDI tertiles. Chi‐squared tests were used for categorical variables and one‐way analysis of variance (ANOVA) tests were used for continuous variables.

We used hurdle models with logistic and negative binomial (log‐link) mixed‐effect regression models for the zero and positive, non‐zero parts of the outcome distribution, respectively, to estimate the associations of SDI with counts of ED visits and non‐elective hospitalizations [40]. The hurdle structure accounts for excess zeros and over‐dispersion commonly present in healthcare utilization data, and the mixed‐effect structure allows for a random effect to account for clustering by HRR. All models were adjusted for the following covariates to control for confounding: sex, age, race, ethnicity, rurality, dual eligibility, cancer type, cancer stage, year of diagnosis, and number of oncology physicians per beneficiary in the HRR. The models were stratified by physician network vulnerability to examine effect modification. To reduce the potential for reverse causality, physician network vulnerability was calculated from the 2016–2017 patient‐sharing networks and outcomes were measured from the 2018–2020 claims. We reported average marginal effect (AME) and accompanying 95% confidence intervals (CI) for the logistic regression component of the hurdle model, and relative risk (RR) and accompanying 95% CIs for the negative binomial mixed‐effect regression component of the hurdle model. The AME was computed as the average difference of the potential outcomes under each level of SDI across the analytic cohort. Statistical significance was designated if the *p* value for a two‐sided hypothesis test was ≤ 0.01; with six outcome analyses (two outcomes by three levels of network vulnerability), this adjustment for multiple comparisons equates to a family‐wise *p* value for the two degree‐of‐freedom test of association of neighborhood deprivation with the outcome of at most 0.06 (a conservative calculation based on the Bonferroni adjustment). All analyses were conducted in R statistical software version 4.3.3.

## 3 Results

We identified 47,756 patients with an incident diagnosis of breast, colorectal or lung cancer in 2018 to 2019 that met our study inclusion criteria. Overall, the study cohort consisted of 19,217 (40.2%) patients with breast cancer, 11,771 (24.6%) patients with colorectal cancer, and 16,768 (35.1%) patients with lung cancer. The majority of patients were female (69.9%), White (88.1%), non‐Hispanic (95.1%), and resided in a metropolitan area (82.4%). The most common cancer stages were stage 1 (47.2%) and stage 4 (22.4%). Nearly one quarter of the cohort had more than two comorbidities (23.0%).

Study cohort characteristics stratified by physician network vulnerability are shown in Table 1. We found that 32.0%, 51.5%, and 16.5% of patients resided in regions of low, medium, and high network vulnerability, respectively. In bivariate analyses between study variables and physician network vulnerability, we observed significant differences (*p* < 0.01) for all demographic, socio‐economic and clinical factors, with the exception of year of diagnosis. Compared to patients in regions with low physician network vulnerability, those in regions with high physician network vulnerability more often resided in micropolitan (30.9% vs. 7.6%) or rural (3.6% vs. 0.8%) areas, were dual‐eligible (18.0% vs. 15.1%.), were of lower socioeconomic status as classified by Yost Index (Quintile 1 (lowest SES): 26.3% vs. 13.8%; Quintile 2: 20.0% vs. 14.4%), and resided in high SDI neighborhoods (51.2% vs. 31.7%).

**TABLE 1:** Characteristics of patients stratified by physician network vulnerability region.

	Physician network vulnerability			*p*
	Low^a^	Medium^b^	High^c^	
*n* (%)^d^	15,281 (32.0)	24,580 (51.5)	7895 (16.5)	
Sex				< 0.001
Male	4637 (30.3)	7200 (29.3)	2547 (32.3)	
Female	10,644 (69.7)	17,380 (70.7)	5348 (67.7)	
Age, mean (SD)	76.34 (7.06)	76.11 (6.97)	75.85 (6.93)	< 0.001
Race				< 0.001
Black	1218 (8.0)	1395 (5.7)	580 (7.3)	
White	13,601 (89.0)	21,446 (87.2)	7011 (88.8)	
Other	462 (3.0)	1739 (7.1)	304 (3.9)	
Ethnicity				< 0.001
Non‐Hispanic	14,694 (96.2)	23,193 (94.4)	7534 (95.4)	
Hispanic	587 (3.8)	1387 (5.6)	361 (4.6)	
Rurality				< 0.001
Metropolitan	14,009 (91.7)	20,155 (82.0)	5172 (65.5)	
Micropolitan	1155 (7.6)	3774 (15.4)	2437 (30.9)	
Rural	117 (0.8)	651 (2.6)	286 (3.6)	
Yost Index Quintile				< 0.001
1 (lowest SES)	2102 (13.8)	2699 (11.0)	2073 (26.3)	
2	2204 (14.4)	4237 (17.2)	1578 (20.0)	
3	2516 (16.5)	5162 (21.0)	1459 (18.5)	
4	3941 (25.8)	5181 (21.1)	1200 (15.2)	
5 (highest SES)	4518 (29.6)	7301 (29.7)	1585 (20.1)	
Social Deprivation Index				< 0.001
Low	5764 (37.7)	6785 (27.6)	1711 (21.7)	
Medium	4675 (30.6)	9498 (38.6)	2143 (27.1)	
High	4842 (31.7)	8297 (33.8)	4041 (51.2)	
Dual‐eligibility				< 0.001
Not dual eligible	12,969 (84.9)	20,595 (83.8)	6471 (82.0)	
Dual eligible	2312 (15.1)	3985 (16.2)	1424 (18.0)	
Number of comorbidities				< 0.001
0	5620 (36.8)	9917 (40.3)	2823 (35.8)	
1	3576 (23.4)	5726 (23.3)	1888 (23.9)	
2	2366 (15.5)	3623 (14.7)	1241 (15.7)	
3+	3719 (24.3)	5314 (21.6)	1943 (24.6)	
Cancer type				< 0.001
Breast cancer	5983 (39.2)	10,258 (41.7)	2976 (37.7)	
Colorectal cancer	3723 (24.4)	6021 (24.5)	2027 (25.7)	
Lung cancer	5575 (36.5)	8301 (33.8)	2892 (36.6)	
Cancer stage				< 0.001
1	7112 (46.5)	11,832 (48.1)	3573 (45.3)	
2	2228 (14.6)	3858 (15.7)	1225 (15.5)	
3	2356 (15.4)	3577 (14.6)	1279 (16.2)	
4	3585 (23.5)	5313 (21.6)	1818 (23.0)	
Year of diagnosis				0.169
2018	7738 (50.6)	12,271 (49.9)	4025 (51.0)	
2019	7543 (49.4)	12,309 (50.1)	3870 (49.0)	
Oncology physician count per beneficiary in HRR, median [IQR]	0.11 [0.08, 0.13]	0.12 [0.10, 0.14]	0.11 [0.11, 0.23]	< 0.001

^a^ In low physician network vulnerability regions, approximately 7% or less of the total oncology physician count was linchpins; 7% represented the first quartile of the distribution of the proportion of linchpin oncology physicians across all HRRs.

^b^ In medium physician network vulnerability regions, greater than 7% and less than approximately 16% of the total oncology physician count were linchpins; 16% represented the median of the distribution of the proportion of linchpin oncology physicians across all HRRs.

^c^ In high physician network vulnerability regions, approximately 16% or greater of the total oncology physician count was linchpins.

^d^ All characteristics show n (%), unless otherwise specified.

Study population characteristics stratified by neighborhood deprivation are shown in Table 2. We found that 29.9%, 34.2%, and 36.0% of patients resided in low, medium, and high SDI neighborhoods, respectively. The bivariate analyses by neighborhood deprivation tertile showed statistically significant differences (*p* < 0.01) for all demographic, socioeconomic, and clinical factors, with the exception of the year of diagnosis. Compared to patients in low SDI neighborhoods, those in high SDI neighborhoods were more often racial and ethnic minorities (Black: 12.9% vs. 2.2%; Hispanic: 7.7% vs. 2.6%), resided in micropolitan (23.7% vs. 7.6%) or rural (3.2% vs. 0.9%) areas, and were more clinically complex (26.8% vs. 19.9% > 2 comorbidities). Additionally, compared to those in the lowest SDI neighborhoods, patients in the highest SDI neighborhoods were more often dual‐eligible (26.1% vs. 8.1%) and resided in high network vulnerability regions (23.5% vs. 12.0%).

**TABLE 2:** Characteristics of patients stratified by neighborhood deprivation tertile.

	Social Deprivation Index (SDI)^a^			*p*
	Low	Medium	High	
*n* (%)^b^	14,260 (29.9)	16,316 (34.2)	17,180 (36)	
Sex				< 0.001
Male	3949 (27.7)	4825 (29.6)	5610 (32.7)	
Female	10,311 (72.3)	11,491 (70.4)	11,570 (67.3)	
Age, mean (SD)	76.58 (7.02)	76.18 (7.03)	75.74 (6.92)	< 0.001
Race				< 0.001
Black	309 (2.2)	676 (4.1)	2208 (12.9)	
White	13,338 (93.5)	14,785 (90.6)	13,935 (81.1)	
Other	613 (4.3)	855 (5.2)	1037 (6.0)	
Ethnicity				< 0.001
Non‐Hispanic	13,886 (97.4)	15,685 (96.1)	15,850 (92.3)	
Hispanic	374 (2.6)	631 (3.9)	1330 (7.7)	
Rurality				< 0.001
Metropolitan	13,050 (91.5)	13,717 (84.1)	12,569 (73.2)	
Micropolitan	1081 (7.6)	2217 (13.6)	4068 (23.7)	
Rural	129 (0.9)	382 (2.3)	543 (3.2)	
Yost Index Quintile				< 0.001
1 (lowest SES)	162 (1.1)	755 (4.6)	5957 (34.7)	
2	494 (3.5)	2585 (15.8)	4940 (28.8)	
3	1584 (11.1)	4237 (26.0)	3316 (19.3)	
4	3598 (25.2)	4693 (28.8)	2031 (11.8)	
5 (highest SES)	8422 (59.1)	4046 (24.8)	936 (5.4)	
Dual‐eligibility				< 0.001
Not dual eligible	13,107 (91.9)	14,238 (87.3)	12,690 (73.9)	
Dual eligible	1153 (8.1)	2078 (12.7)	4490 (26.1)	
Number of comorbidities				< 0.001
0	6128 (43.0)	6478 (39.7)	5754 (33.5)	
1	3271 (22.9)	3849 (23.6)	4070 (23.7)	
2	2025 (14.2)	2448 (15.0)	2757 (16.0)	
3+	2836 (19.9)	3541 (21.7)	4599 (26.8)	
Cancer type				< 0.001
Breast cancer	6168 (43.3)	6717 (41.2)	6332 (36.9)	
Colorectal cancer	3373 (23.7)	3891 (23.8)	4507 (26.2)	
Lung cancer	4719 (33.1)	5708 (35.0)	6341 (36.9)	
Cancer stage				< 0.001
1	7248 (50.8)	7865 (48.2)	7404 (43.1)	
2	2086 (14.6)	2517 (15.4)	2708 (15.8)	
3	1937 (13.6)	2412 (14.8)	2863 (16.7)	
4	2989 (21.0)	3522 (21.6)	4205 (24.5)	
Year of diagnosis				0.044
2018	7232 (50.7)	8287 (50.8)	8515 (49.6)	
2019	7028 (49.3)	8029 (49.2)	8665 (50.4)	
Physician network vulnerability				< 0.001
Low	5764 (40.4)	4675 (28.7)	4842 (28.2)	
Medium	6785 (47.6)	9498 (58.2)	8297 (48.3)	
High	1711 (12.0)	2143 (13.1)	4041 (23.5)	
Oncology physician count per beneficiary in HRR, median [IQR]	0.12 [0.09, 0.13]	0.12 [0.10, 0.14]	0.11 [0.09, 0.13]	< 0.001

^a^ The SDI score threshold between the low and medium categories was 31 and 36 for 2018 and 2019, respectively. The SDI score threshold between medium and high categories was 61 and 60 for 2018 and 2019, respectively.

^b^ All characteristics show *n* (%), unless otherwise specified.

Figure S4 shows the proportion of the analytic cohort with at least one ED visit and one non‐elective hospitalization during the first 12 months after their initial cancer diagnosis by physician network vulnerability region. Of patients residing in low, medium, and high physician network vulnerability HRRs, 40.6%, 40.9%, and 45.3% had at least one ED visit and 39.4%, 36.5%, and 39.5% had at least one non‐elective hospitalization, respectively.

**FIGURE 2:**
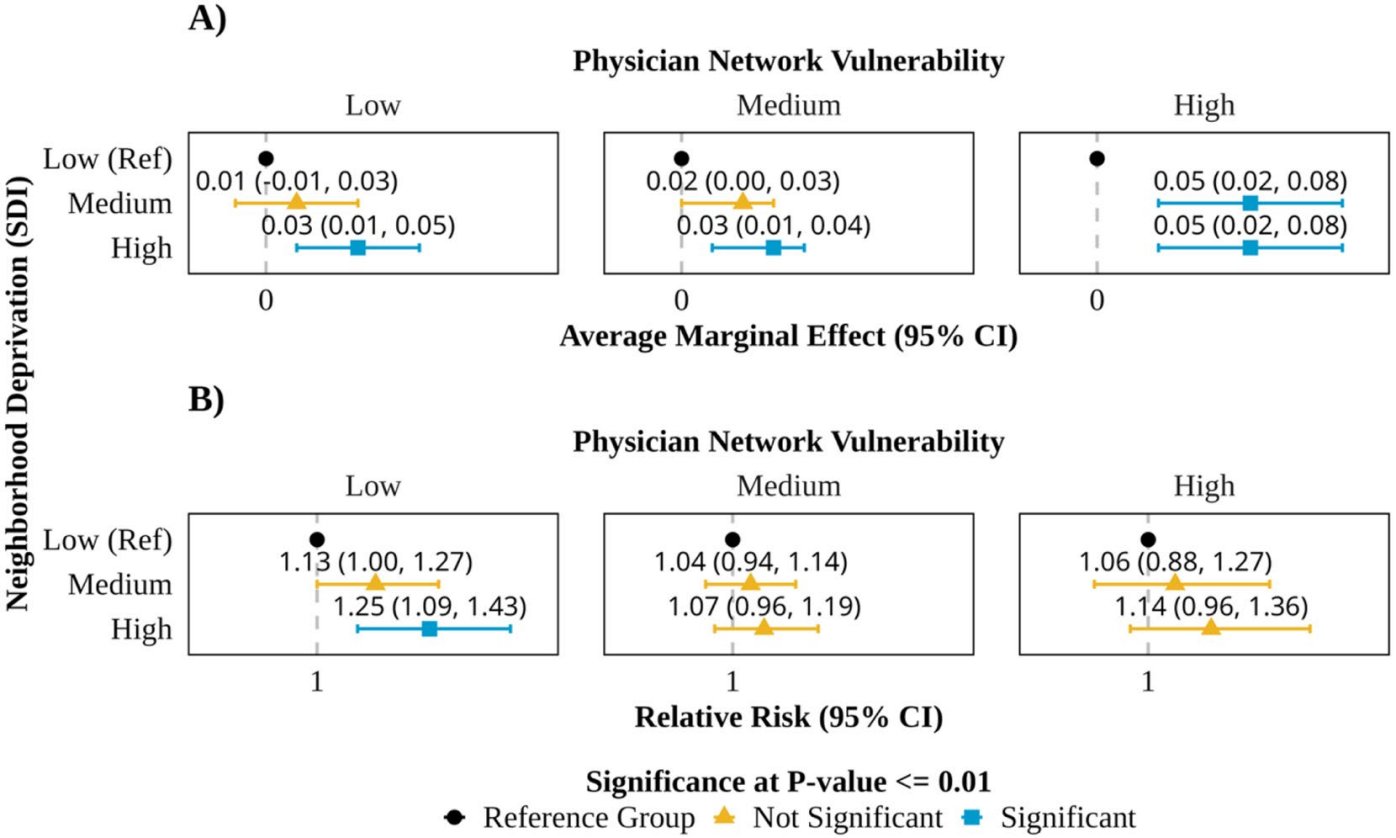
Main effect results from the hurdle model where the outcome was emergency department visits and the effect modifier was physician network vulnerability. (A) Results from the zero component (i.e., logistic regression) of the hurdle model that shows the association between neighborhood deprivation (SDI) and the average change in probability of having at least one emergency department visit vs. none. The statistically significant average marginal effect (AME) results are also statistically significant on the odds‐ratio scale. AME is reported for easier interpretation. (B) Results from the positive, non‐zero component (i.e., negative binomial regression) of the hurdle model that shows the association between neighborhood deprivation (SDI) and the relative risk of additional emergency department visits, conditional on at least one visit. SDI: social deprivation index.

The results from the hurdle regression that modeled ER visits are presented in Figure 2. The probability of at least one ED visit was higher among patients in high SDI neighborhoods compared to those in low SDI neighborhoods across all physician network vulnerability strata. In the low and medium network vulnerability strata, patients had similar increases in the probability of at least one ED visit when they resided in a high SDI neighborhood (low network vulnerability—AME [95% CI]: 0.03 [0.01–0.05]; medium network vulnerability—AME [95% CI]: 0.03 [0.01–0.04]). In the high network vulnerability stratum, patients living in both medium and high SDI had increased probability of at least one ED visit (medium SDI—AME [95% CI]: 0.05 [0.02–0.08]; high SDI—AME [95% CI]: 0.05 [0.02–0.08]). Conditional on having at least one ED visit, we observed greater relative risk of additional ED visits for patients in high SDI neighborhoods compared with those in low SDI neighborhoods in regions characterized as low physician network vulnerability (RR [95% CI]: 1.25 [1.09–1.43]). In regions characterized as medium or high physician network vulnerability, there were no statistically significant associations between SDI and the rate of ED visits. Table S1 includes fully adjusted model results (zero and positive, non‐zero components) for ED visits.

**FIGURE 3:**
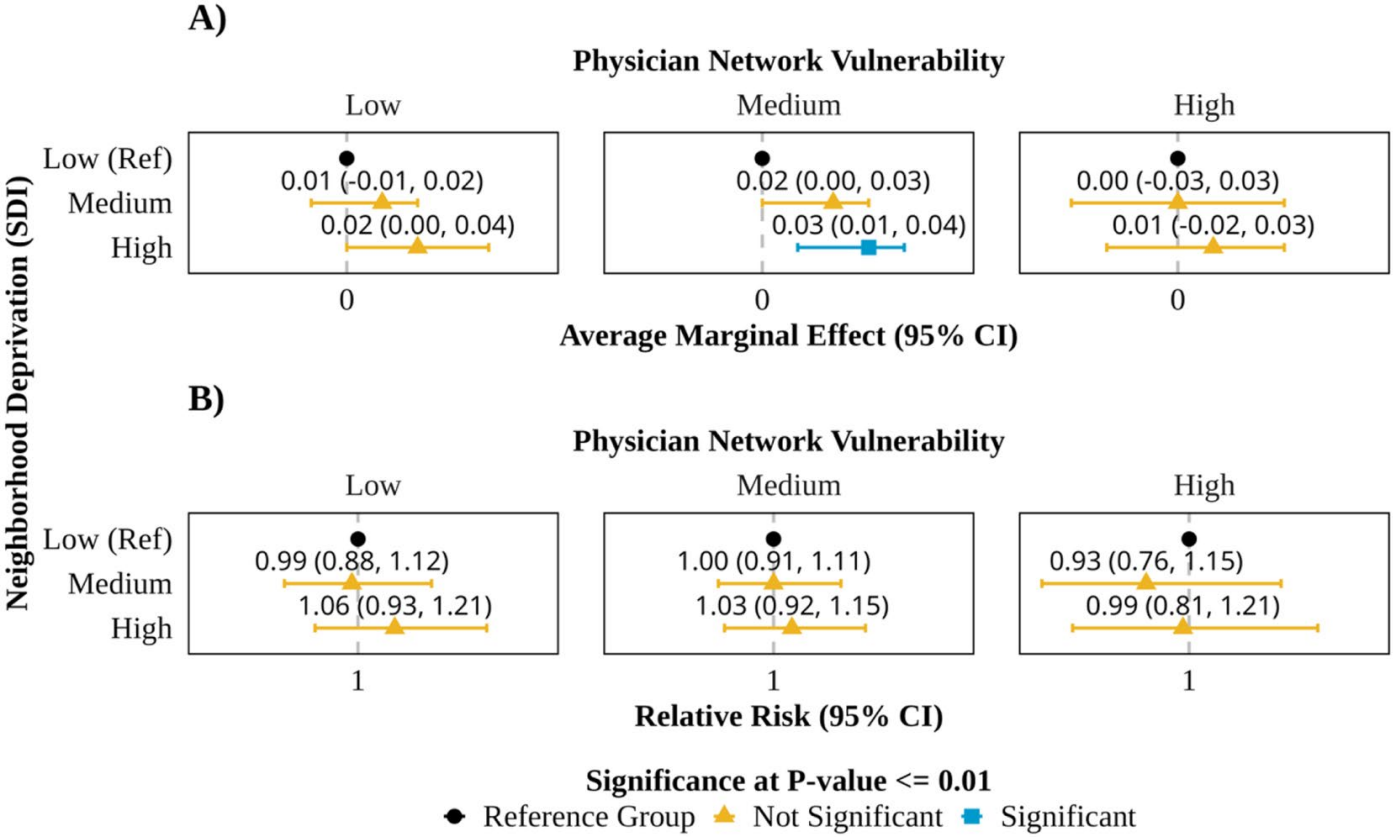
Main effect results from the hurdle model where the outcome was non‐elective hospitalizations and the effect modifier was physician network vulnerability. (A) Results from the zero component (i.e., logistic regression) of the hurdle model that shows the association between neighborhood deprivation (SDI) and the average change in probability of having at least one non‐elective hospitalization vs. none. The statistically significant average marginal effect (AME) results are also statistically significant on the odds‐ratio scale. AME is reported for easier interpretation. (B) Results from the positive, non‐zero component (i.e., negative binomial regression) of the hurdle model that shows the association between neighborhood deprivation (SDI) and the relative risk of additional non‐elective hospitalizations, conditional on at least one. SDI: social deprivation index.

The results from the hurdle regression that modeled non‐elective hospitalizations are presented in Figure 3. The probability of at least one non‐elective hospitalization was higher among patients in high, compared to low, SDI neighborhoods in regions described as medium physician network vulnerability (AME [95% CI]: 0.03 [0.01–0.04]). There were no other significant associations with the probability of at least one non‐elective hospitalization. Additionally, we did not find any significant associations between SDI and the rate of non‐elective hospitalizations across any of the physician network vulnerability strata. Table S2 include fully adjusted model results (zero and positive, non‐zero components) for non‐elective hospitalizations.

## 4 Discussion

In this study, we evaluated the associations of social deprivation with ED visits and non‐elective hospitalizations after cancer diagnosis, and we examined effect modification of physician network vulnerability. We found a statistically significant association between greater neighborhood social deprivation and a higher probability of having at least one ED visit across all physician network vulnerability strata. Although subtle, our findings show that the highest probability of having at least one ED visit was among patients with medium or high neighborhood SDI who are also residing in regions with high physician network vulnerability. Contingent on at least one ED visit, our models found a moderately increased relative risk of ED visits only among patients living in regions with low physician network vulnerability. We also found a statistically significant association between greater neighborhood social deprivation and a higher probability of having at least one non‐elective hospitalization among patients living in regions with medium physician network vulnerability; however, social deprivation was not associated with the rate of non‐elective hospitalizations in any stratum of physician network vulnerability. Novel contributions of this work include our characterization of a SEER‐Medicare patient cohort by neighborhood SDI, and our examination of how SDI was associated with acute care utilization in different oncology scarcity settings among patients with cancer.

We hypothesized that the association of neighborhood deprivation with acute care use would be positive and modified by physician network vulnerability; specifically, that differences in acute care use by neighborhood SDI would be exacerbated in high physician network vulnerability regions and mitigated in low physician network vulnerability regions. In support of our hypothesis, we found that the likelihood of having at least one ED visit was greatest in high SDI neighborhoods compared to low SDI neighborhoods, and that the effect of living in a medium or high SDI neighborhood on the probability of having at least one ED visit was greatest in regions characterized by high physician network vulnerability. This suggests that strengthening or expanding relationships with oncology physicians to reduce vulnerabilities (i.e., decrease reliance on linchpin oncologists) in areas with medium or high SDI may have a modest protective effect on the probability of ED visits during treatment. However, these findings were not mirrored in the positive, non‐zero component of the ED visit hurdle model or in either component of the non‐elective hospitalization hurdle model.

One potential explanation for the mostly null interactions from the positive, non‐zero components observed for both study outcomes is that physician network vulnerability, used in this study as a measure of oncologist scarcity, has little impact on the relatively crude measures of acute care use that we studied. Prior work studying measures of the oncology workforce, such as oncologist density or physician network vulnerability, evaluated cancer‐specific outcomes, including stage at diagnosis, multidisciplinary cancer consultations, chemotherapy receipt, or cancer mortality rates [28, 41, 42]. We used physician network vulnerability as a proxy for access to multidisciplinary cancer care, so it is possible that a lack of access to multidisciplinary cancer care as captured by this measure is not a contributing factor to all‐cause acute care utilization. Another possibility is that the outcomes of ED visits and non‐elective hospitalizations may be too heterogeneous to adequately capture the effects of multidisciplinary cancer care, since ED visits and non‐elective hospitalizations can have highly disparate causes and patient impacts.

We hypothesized significant interactions of neighborhood deprivation and physician network vulnerability because health care resources associated with low physician network vulnerability regions may help alleviate some of the barriers to care experienced by patients living in socially deprived neighborhoods. In fact, we found that these multilevel vulnerabilities were strongly correlated with each other. In regions characterized as low physician network vulnerability, the majority of patients also resided in the lowest SDI neighborhoods, whereas in regions characterized as high physician network vulnerability, most patients also resided in the highest SDI neighborhoods. These correlations demonstrate how patients experience vulnerabilities at multiple socioecological levels at once. Our findings indicate that the interaction between individual‐, neighborhood‐, and health system‐level factors plays some role in patients' access to and use of care. Potential interventions to address social drivers of health are integrating healthcare professionals such as patient navigators, social workers, and community health workers into cancer care teams for patients facing multilevel social vulnerabilities.

Given the numerous measures that have been created to study SDOH, clarity and guidance on how to select appropriate measures for specific studies may enhance the reproducibility of findings. For this study, we used SDI because it was explicitly developed to study differences in health outcomes by neighborhood SES and the mapping of census tract SDI scores to patient ZIP codes was novel, but there are many other area‐level SES measures used in cancer health services research. A scoping review on this topic found 18 different indices used across 45 studies, but that ADI, SVI, and the NCI SES index were the most common [43]. Notably, these measures are oftentimes associated with each other. In our study, we found that SDI and the Yost Index were strongly correlated. Figure S5 shows that as the Yost Index increases from lowest SES to highest SES, the proportion of patients in high SDI neighborhoods drastically decreases from 86.7% to 7%. It is important to consider the following when deciding which neighborhood‐level SES index to use. First, what geographic unit is preferred and is the data linkage feasible? Second, what specific social risks are relevant to the research question and are they reflected in the composite index? Third, are the years of data used to create the area‐level SES measure consistent with the years of other data sources in the study? The area‐level SES measures are maintained by various organizations and institutions, and the extent to which they are updated to reflect current years could influence their utility in future studies.

## 5 Limitations

This study has several limitations. First, our study cohort consisted of patients with breast, colorectal and lung cancer older than 66 years, which means our findings may not be generalizable to other cancer types or non‐Medicare populations. Second, we used HRRs to represent large health systems, which may not reflect cancer care delivery systems. Third, given the retrospective observational study design, we cannot claim causality of any findings. Fourth, there is a risk of residual confounding from unavailable or unmeasured patient‐, neighborhood‐, or health system‐level factors, and we do not have individual‐level measures of social deprivation. Our results are least vulnerable to health system‐level unmeasured confounding due to the inclusion of HRR as a random effect in the hurdle models.

## 6 Conclusions

In this retrospective cohort study of SEER‐Medicare data, we evaluated how individual‐, neighborhood‐, and health system‐level drivers of health influenced acute care use following cancer diagnosis. We found significant associations between neighborhood SDI and the probability of having at least one ED visit, and that the effect was strongest in regions with high network vulnerability. More research is needed to understand how multilevel socioecological vulnerabilities interact to influence outcomes across the cancer care continuum.

## Ethics Statement

The authors have nothing to report.

## Conflicts of Interest

The authors declare no conflicts of interest.

## Supporting information


**Data S1:** hesr70070‐sup‐0001‐Supinfo.docx.

## Data Availability

The data that support the findings of this study are available from NCI. Restrictions apply to the availability of these data, which were used under license for this study. Researchers need to apply to access the data through NCI.
