# Supplementary material for: Associations of Social Deprivation and Oncology Physician Network Vulnerability With Acute Care Utilization in the SEER‐Medicare Population
Source: Health Serv Res. 2025 Nov 18;61(1):e70070. doi: 10.1111/1475-6773.70070 (PMC12807442; doi:10.1111/1475-6773.70070)
Supplement: Supplementary file 1 — Data S1: hesr70070‐sup‐0001‐Supinfo.docx. [file HESR-61-e70070-s001.docx]

Associations of Social Deprivation and Oncology Physician Network Vulnerability with Acute Care Utilization in the SEER-Medicare Population

**Supplementary Material**

**Supplemental Figure 1.** Flow diagram of selection of patient analytic cohort.

**Supplemental Figure 2.** Flow diagram of selection of providers used in patient-sharing network analyses (2016-2017).

**Supplemental Figure 3.** Example of how to calculate linchpin score from a patient-sharing network and how linchpin score is aggregated up to HRR physician network vulnerability.

**Supplemental Table 1.** Fully adjusted results from the hurdle model where the outcome was emergency department visits and the effect modifier was physician network vulnerability.

**Supplemental Table 2.** Fully adjusted results from the hurdle model where the outcome was non-elective hospitalizations and the effect modifier was physician network vulnerability.

**Supplemental Table 3.** Fully adjusted results from two hurdle models where one outcome was emergency department visits and one oncome was non-elective hospitalizations, and the interaction term was for social deprivation and physician network vulnerability.

**Supplemental Figure 4.** Proportion of the analytic cohort that had at least one emergency department visit and/or at least one non-elective hospitalization during the first 12 months after their initial cancer diagnosis, stratified by physician network vulnerability region.

**Supplemental Figure 5.** Association between Social Deprivation Index (SDI) and Yost Index quintile among the analytic cohort.

**Supplemental Figure 1.** Flow diagram of selection of patient analytic cohort.

1. Patients with an incident cancer diagnosis in 2016 – 2017 were used to form the HRR-level patient-sharing networks.

2. We created a patient-sharing network from 2016 – 2017 Part B claims and subsequently derived Physician Network Vulnerability from this patient-sharing network. This 2016 – 2017 patient-sharing network included patients with an incident cancer diagnosis in 2016 – 2017.

3. Patients with unknown/unassigned cancer stage were predominantly from registries that did not report AJCC cancer stage.

MBSF: Medicare Beneficiary Summary File.

AJCC: American Joint Committee on Cancer.

**Supplemental Figure 2.** Flow diagram of selection of providers used in patient-sharing network analyses (2016-2017).

* The providers in the final 2016 – 2017 patient-sharing network were used to calculate linchpin score, network vulnerability, and oncology physician count per beneficiary covariate in the hurdle models.

HRR: hospital referral region.

**Supplemental Figure 3.** Example of how to calculate linchpin score from a patient-sharing network and how linchpin score is aggregated up to HRR physician network vulnerability.

Abbreviations: med oncs – medical oncologists; rad oncs – radiation oncologists; surg oncs – surgical oncologists.

**Supplemental Table 1.** Fully adjusted results from the hurdle model where the outcome was emergency department visits and the effect modifier was physician network vulnerability.

|  | **Zero Component**  (Probability of at least one visit vs. no visits) | | | **Positive, Non-zero Component**  (Relative risk of additional visits conditional on at least one) | | |
| --- | --- | --- | --- | --- | --- | --- |
|  | Physician Network Vulnerability | | | Physician Network Vulnerability | | |
|  | Low | Medium | High | Low | Medium | High |
|  | Average Marginal  Effect  (95% CI) | Average  Marginal  Effect  (95% CI) | Average  Marginal  Effect  (95% CI) | Relative Risk (95% CI) | Relative Risk (95% CI) | Relative Risk (95% CI) |
| *Social Deprivation Index* |  |  |  |  |  |  |
| Low | Ref-- | Ref-- | Ref-- | Ref-- | Ref-- | Ref-- |
| Medium | 0.01  (-0.01, 0.03) | 0.02  (0.00, 0.03) | 0.05  (0.02, 0.08) * | 1.13  (1.00, 1.27) | 1.04  (0.94, 1.14) | 1.06  (0.88, 1.27) |
| High | 0.03  (0.01, 0.05) * | 0.03  (0.01, 0.04) * | 0.05  (0.02, 0.08) * | 1.25  (1.09, 1.43) * | 1.07  (0.96, 1.19) | 1.14  (0.96, 1.36) |
| *Sex* |  |  |  |  |  |  |
| Male | Ref-- | Ref-- | Ref-- | Ref-- | Ref-- | Ref-- |
| Female | -0.01  (-0.03, 0.01) | 0.00  (-0.02, 0.01) | -0.01  (-0.03, 0.02) | 1.01  (0.91, 1.13) | 0.90  (0.82, 0.98) | 0.87  (0.76, 1.00) |
| *Age* | 0.00  (0.00, 0.00) * | 0.00  (0.00, 0.01) * | 0.00  (0.00, 0.01) * | 1.00  (0.99, 1.01) | 1.00  (1.00, 1.01) | 1.00  (1.00, 1.01) |
| *Race* |  |  |  |  |  |  |
| White | Ref-- | Ref-- | Ref-- | Ref-- | Ref-- | Ref-- |
| American Indian Alaskan Native | 0.06  (-0.04, 0.16) | 0.10  (0.02, 0.18) | 0.18  (0.02, 0.35) | 1.84  (1.07, 3.14) | 1.30  (0.83, 2.04) | 1.38  (0.61, 3.15) |
| Asian & Pacific Islander | -0.08  (-0.12, -0.03) * | -0.08  (-0.10, -0.06) * | -0.10  (-0.15, -0.04) * | 0.77  (0.55, 1.08) | 0.75  (0.63, 0.89) * | 0.61  (0.41, 0.90) |
| Black | 0.04  (0.01, 0.07) * | 0.06  (0.03, 0.09) * | 0.07  (0.03, 0.11) * | 1.15  (0.97, 1.35) | 1.30  (1.12, 1.51) * | 1.25  (1.01, 1.55) |
| *Ethnicity* |  |  |  |  |  |  |
| Non-Hispanic | Ref-- | Ref-- | Ref-- | Ref-- | Ref-- | Ref-- |
| Hispanic | 0.04  (0.00, 0.08) | 0.01  (-0.01, 0.04) | 0.04  (-0.01, 0.10) | 1.17  (0.93, 1.46) | 1.24  (1.06, 1.47) * | 0.95  (0.72, 1.25) |
| *Rurality-Urbanicity* |  |  |  |  |  |  |
| Metropolitan | Ref-- | Ref-- | Ref-- | Ref-- | Ref-- | Ref-- |
| Micropolitan | 0.03  (0.00, 0.06) | 0.04  (0.02, 0.06) * | 0.04  (0.01, 0.07) * | 1.02  (0.85, 1.22) | 1.12  (1.00, 1.25) | 1.24  (1.08, 1.42) * |
| Rural | 0.07  (-0.02, 0.15) | 0.04  (0.00, 0.08) | -0.02  (-0.07, 0.04) | 1.12  (0.67, 1.86) | 0.99  (0.78, 1.24) | 0.84  (0.61, 1.17) |
| *Dual-eligible* |  |  |  |  |  |  |
| Not Dual Eligible | Ref-- | Ref-- | Ref-- | Ref-- | Ref-- | Ref-- |
| Dual Eligible | 0.09  (0.07, 0.12) * | 0.10  (0.08, 0.11) * | 0.10  (0.07, 0.13) * | 1.54  (1.36, 1.73) * | 1.39  (1.26, 1.54) * | 1.38  (1.19, 1.59) * |
| *Number of Comorbidities* |  |  |  |  |  |  |
| 0 | Ref-- | Ref-- | Ref-- | Ref-- | Ref-- | Ref-- |
| 1 | 0.05  (0.03, 0.07) * | 0.06  (0.05, 0.08) * | 0.06  (0.03, 0.08) * | 1.27  (1.12, 1.45) * | 1.19  (1.07, 1.32) * | 1.23  (1.04, 1.46) |
| 2 | 0.10  (0.08, 0.12) * | 0.10  (0.08, 0.11) * | 0.07  (0.04, 0.11) * | 1.41  (1.22, 1.62) * | 1.37  (1.22, 1.54) * | 1.68  (1.40, 2.01) * |
| 3+ | 0.15  (0.13, 0.17) * | 0.15  (0.14, 0.17) * | 0.14  (0.11, 0.17) * | 1.61  (1.42, 1.82) * | 1.69  (1.53, 1.88) * | 2.17  (1.85, 2.54) * |
| *Cancer Type* |  |  |  |  |  |  |
| Breast Cancer | Ref-- | Ref-- | Ref-- | Ref-- | Ref-- | Ref-- |
| Colorectal Cancer | 0.06  (0.04, 0.09) * | 0.06  (0.04, 0.07) * | 0.04  (0.01, 0.07) | 1.26  (1.09, 1.45) * | 1.31  (1.17, 1.47) * | 1.02  (0.85, 1.22) |
| Lung Cancer | 0.13  (0.11, 0.15) * | 0.13  (0.11, 0.15) * | 0.14  (0.11, 0.17) * | 1.63  (1.43, 1.87) * | 1.62  (1.45, 1.80) * | 1.23  (1.04, 1.46) |
| *Cancer Stage* |  |  |  |  |  |  |
| 1 | Ref-- | Ref-- | Ref-- | Ref-- | Ref-- | Ref-- |
| 2 | 0.08  (0.05, 0.10) * | 0.08  (0.06, 0.10) * | 0.05  (0.02, 0.08) * | 1.31  (1.14, 1.51) * | 1.29  (1.15, 1.44) * | 0.99  (0.82, 1.19) |
| 3 | 0.14  (0.11, 0.16) * | 0.14  (0.12, 0.16) * | 0.13  (0.10, 0.16) * | 1.32  (1.15, 1.52) * | 1.46  (1.30, 1.63) * | 1.34  (1.12, 1.59) * |
| 4 | 0.26  (0.24, 0.28) * | 0.26  (0.24, 0.28) * | 0.27  (0.24, 0.30) * | 1.85  (1.62, 2.10) * | 2.08  (1.87, 2.31) * | 2.37  (2.01, 2.78) * |
| *Year of Diagnosis* |  |  |  |  |  |  |
| 2018 | Ref-- | Ref-- | Ref-- | Ref-- | Ref-- | Ref-- |
| 2019 | -0.01  (-0.02, 0.01) | -0.02  (-0.03, -0.01) * | -0.03  (-0.05, -0.01) * | 0.90  (0.82, 0.99) | 0.95  (0.88, 1.02) | 0.92  (0.82, 1.03) |
| *Oncology Physician Count*  *Per Beneficiary in HRR* | 0.02  (-0.28, 0.32) | -0.13  (-0.37, 0.11) | -0.01  (-0.04, 0.02) | 0.75  (0.12, 4.69) | 0.39  (0.07, 2.36) | 1.03  (0.87, 1.23) |
| *Medicare Advantage*  *Penetration Rate in*  *Patient's State of Residence* | 0.03  (-0.09, 0.14) | -0.08  (-0.18, 0.03) | -0.08  (-0.22, 0.07) | 0.73  (0.37, 1.42) | 0.94  (0.42, 2.10) | 0.48  (0.26, 0.88) |

* Statistically significant at p-value <= 0.01.

**Supplemental Table 2.** Fully adjusted results from the hurdle model where the outcome was non-elective hospitalizations and the effect modifier was physician network vulnerability.

|  | **Zero Component**  (Probability of at least one visit vs. no visits) | | | **Positive, Non-zero Component**  (Relative risk of additional visits conditional on at least one) | | |
| --- | --- | --- | --- | --- | --- | --- |
|  | Physician Network Vulnerability | | | Physician Network Vulnerability | | |
|  | Low | Medium | High | Low | Medium | High |
|  | Average  Marginal Effect  (95% CI) | Average  Marginal Effect  (95% CI) | Average  Marginal Effect  (95% CI) | Relative Risk (95% CI) | Relative Risk (95% CI) | Relative Risk (95% CI) |
| *Social Deprivation Index* |  |  |  |  |  |  |
| Low | Ref-- | Ref-- | Ref-- | Ref-- | Ref-- | Ref-- |
| Medium | 0.01  (-0.01, 0.02) | 0.02  (0.00, 0.03) | 0.00  (-0.03, 0.03) | 0.99  (0.88, 1.12) | 1.00  (0.91, 1.11) | 0.93  (0.76, 1.15) |
| High | 0.02  (0.00, 0.04) | 0.03  (0.01, 0.04) * | 0.01  (-0.02, 0.03) | 1.06  (0.93, 1.21) | 1.03  (0.92, 1.15) | 0.99  (0.81, 1.21) |
| *Sex* |  |  |  |  |  |  |
| Male | Ref-- | Ref-- | Ref-- | Ref-- | Ref-- | Ref-- |
| Female | -0.02  (-0.04, -0.01) * | -0.02  (-0.04, -0.01) * | -0.02  (-0.05, 0.00) | 0.96  (0.87, 1.06) | 0.92  (0.84, 1.00) | 1.09  (0.94, 1.26) |
| *Age* | 0.00  (0.00, 0.01) * | 0.01  (0.00, 0.01) * | 0.01  (0.00, 0.01) * | 1.00  (0.99, 1.00) | 1.00  (0.99, 1.01) | 1.00  (0.99, 1.01) |
| *Race* |  |  |  |  |  |  |
| White | Ref-- | Ref-- | Ref-- | Ref-- | Ref-- | Ref-- |
| American Indian Alaskan Native | 0.10  (0.01, 0.19) | 0.08  (0.00, 0.15) | 0.12  (-0.04, 0.27) | 1.18  (0.69, 2.04) | 1.26  (0.76, 2.07) | 1.54  (0.59, 4.03) |
| Asian & Pacific Islander | -0.05  (-0.10, -0.01) | -0.06  (-0.08, -0.04) * | -0.12  (-0.17, -0.07) * | 0.80  (0.58, 1.11) | 0.96  (0.82, 1.14) | 0.60  (0.39, 0.92) |
| Black | 0.01  (-0.01, 0.04) | 0.00  (-0.02, 0.03) | 0.06  (0.02, 0.10) * | 1.04  (0.88, 1.22) | 1.19  (1.01, 1.39) | 1.27  (1.00, 1.62) |
| *Ethnicity* |  |  |  |  |  |  |
| Non-Hispanic | Ref-- | Ref-- | Ref-- | Ref-- | Ref-- | Ref-- |
| Hispanic | -0.06  (-0.09, -0.02) * | -0.01  (-0.04, 0.01) | 0.02  (-0.03, 0.06) | 0.97  (0.75, 1.25) | 1.04  (0.87, 1.23) | 0.83  (0.61, 1.14) |
| *Rurality-Urbanicity* |  |  |  |  |  |  |
| Metropolitan | Ref-- | Ref-- | Ref-- | Ref-- | Ref-- | Ref-- |
| Micropolitan | -0.04  (-0.07, -0.01) * | -0.03  (-0.04, -0.01) * | 0.00  (-0.03, 0.02) | 0.99  (0.81, 1.21) | 0.92  (0.81, 1.04) | 1.14  (0.95, 1.36) |
| Rural | -0.06  (-0.15, 0.02) | -0.03  (-0.06, 0.00) | -0.04  (-0.09, 0.01) | 0.91  (0.49, 1.71) | 0.92  (0.71, 1.18) | 0.84  (0.57, 1.23) |
| *Dual-eligible* |  |  |  |  |  |  |
| Not Dual Eligible | Ref-- | Ref-- | Ref-- | Ref-- | Ref-- | Ref-- |
| Dual Eligible | 0.09  (0.07, 0.11) * | 0.08  (0.07, 0.10) * | 0.09  (0.06, 0.12) * | 1.36  (1.21, 1.53) * | 1.19  (1.08, 1.32) * | 1.27  (1.08, 1.49) * |
| *Number of Comorbidities* |  |  |  |  |  |  |
| 0 | Ref-- | Ref-- | Ref-- | Ref-- | Ref-- | Ref-- |
| 1 | 0.05  (0.03, 0.07) * | 0.05  (0.04, 0.07) * | 0.05  (0.03, 0.08) * | 1.22  (1.07, 1.40) * | 1.14  (1.02, 1.27) | 1.22  (1.00, 1.47) |
| 2 | 0.10  (0.08, 0.12) * | 0.08  (0.06, 0.10) * | 0.08  (0.05, 0.11) * | 1.32  (1.14, 1.53) * | 1.30  (1.15, 1.47) * | 1.42  (1.15, 1.75) * |
| 3+ | 0.20  (0.18, 0.21) * | 0.17  (0.16, 0.19) * | 0.18  (0.15, 0.20) * | 1.84  (1.62, 2.08) * | 1.72  (1.55, 1.92) * | 2.05  (1.72, 2.45) * |
| *Cancer Type* |  |  |  |  |  |  |
| Breast Cancer | Ref-- | Ref-- | Ref-- | Ref-- | Ref-- | Ref-- |
| Colorectal Cancer | 0.16  (0.14, 0.18) * | 0.15  (0.14, 0.17) * | 0.15  (0.12, 0.18) * | 1.36  (1.17, 1.58) * | 1.37  (1.21, 1.56) * | 1.25  (1.01, 1.54) |
| Lung Cancer | 0.22  (0.20, 0.24) * | 0.19  (0.18, 0.21) * | 0.18  (0.15, 0.21) * | 1.80  (1.56, 2.08) * | 1.84  (1.63, 2.08) * | 1.89  (1.54, 2.31) * |
| *Cancer Stage* |  |  |  |  |  |  |
| 1 | Ref-- | Ref-- | Ref-- | Ref-- | Ref-- | Ref-- |
| 2 | 0.13  (0.11, 0.15) * | 0.11  (0.10, 0.13) * | 0.10  (0.07, 0.12) * | 1.31  (1.12, 1.52) * | 1.29  (1.14, 1.47) * | 1.22  (0.98, 1.51) |
| 3 | 0.21  (0.19, 0.24) * | 0.21  (0.20, 0.23) * | 0.22  (0.19, 0.26) * | 1.46  (1.27, 1.67) * | 1.51  (1.34, 1.70) * | 1.54  (1.27, 1.88) * |
| 4 | 0.42  (0.40, 0.44) * | 0.44  (0.42, 0.45) * | 0.43  (0.40, 0.45) * | 2.47  (2.18, 2.81) * | 2.43  (2.18, 2.71) * | 2.61  (2.18, 3.14) * |
| *Year of Diagnosis* |  |  |  |  |  |  |
| 2018 | Ref-- | Ref-- | Ref-- | Ref-- | Ref-- | Ref-- |
| 2019 | 0.00  (-0.02, 0.01) | -0.02  (-0.03, -0.01) * | -0.02  (-0.04, 0.00) | 1.03  (0.94, 1.13) | 0.93  (0.86, 1.01) | 0.96  (0.84, 1.09) |
| *Oncology Physician Count Per Beneficiary in HRR* | -0.10  (-0.53, 0.33) | -0.23  (-0.47, 0.01) | -0.01  (-0.04, 0.02) | 1.02  (0.13, 8.04) | 0.77  (0.17, 3.49) | 1.07  (0.88, 1.29) |
| *Medicare Advantage*  *Penetration Rate in*  *Patient's State of Residence* | -0.04  (-0.21, 0.13) | -0.07  (-0.17, 0.04) | 0.01  (-0.13, 0.15) | 0.97  (0.43, 2.21) | 0.79  (0.44, 1.42) | 0.67  (0.20, 2.23) |

* Statistically significant at p-value <= 0.01.

**Supplemental Table 3.** Fully adjusted results from two hurdle models where one outcome was emergency department visits and one oncome was non-elective hospitalizations, and the interaction term was for social deprivation and physician network vulnerability.

|  | **Emergency Department Visits** | | **Non-elective Hospitalizations** | |
| --- | --- | --- | --- | --- |
|  | Zero Component^a^ | Conditional Component^b^ | Zero Component^a^ | Conditional Component^b^ |
|  | Average Marginal Effect (95% CI) | Relative Risk  (95% CI) | Average Marginal Effect (95% CI) | Relative Risk  (95% CI) |
| *Social Deprivation Index (SDI)* |  |  |  |  |
| Low | Ref-- | Ref-- | Ref-- | Ref-- |
| Medium | 0.02  (0.01, 0.03) * | 1.13  (1.00, 1.28) | 0.01  (0.00, 0.02) | 1.00  (0.88, 1.12) |
| High | 0.03  (0.02, 0.04) * | 1.24  (1.09, 1.40) * | 0.02  (0.01, 0.03) * | 1.06  (0.93, 1.20) |
| Physician Network Vulnerability |  |  |  |  |
| Low | Ref-- | Ref-- | Ref-- | Ref-- |
| Medium | 0.03  (0.01, 0.05) * | 1.18  (1.02, 1.36) | 0.01  (-0.02, 0.03) | 0.98  (0.85, 1.14) |
| High | 0.01  (0.00, 0.03) | 1.16  (0.95, 1.41) | 0.00  (-0.02, 0.02) | 1.06  (0.87, 1.30) |
| *SDI x Physician Network Vulnerability* |  |  |  |  |
| Medium SDI x Medium Network Vulnerability | ----- | 0.92  (0.74, 1.02) | ----- | 1.00  (0.82, 1.13) |
| High SDI x Medium Network Vulnerability | ----- | 0.87  (0.73, 1.15) | ----- | 0.96  (0.74, 1.17) |
| Medium SDI x High Network Vulnerability | ----- | 0.92  (0.76, 1.19) | ----- | 0.93  (0.77, 1.20) |
| High SDI x High Network Vulnerability | ----- | 0.95  (1.12, 1.23) | ----- | 0.96  (1.13, 1.24) |
| *Sex* |  |  |  |  |
| Male | Ref-- | Ref-- | Ref-- | Ref-- |
| Female | -0.01  (-0.02, 0.00) | 0.93  (0.87, 0.99) | -0.02  (-0.03, -0.01) * | 0.96  (0.91, 1.02) |
| *Age* | 0.00  (0.00, 0.00) * | 1.00  (1.00, 1.01) | 0.01  (0.00, 0.01) * | 1.00  (1.00, 1.00) |
| *Race* |  |  |  |  |
| White | Ref-- | Ref-- | Ref-- | Ref-- |
| American Indian Alaskan Native | 0.10  (0.04, 0.16) * | 1.47  (1.07, 2.03) | 0.09  (0.04, 0.15) * | 1.27  (0.90, 1.80) |
| Asian & Pacific Islander | -0.08  (-0.10, -0.06) * | 0.73  (0.63, 0.84) * | -0.07  (-0.08, -0.05) * | 0.88  (0.77, 1.01) |
| Black | 0.06  (0.04, 0.07) * | 1.22  (1.11, 1.35) * | 0.02  (0.00, 0.03) | 1.14  (1.03, 1.27) |
| *Ethnicity* |  |  |  |  |
| Non-Hispanic | Ref-- | Ref-- | Ref-- | Ref-- |
| Hispanic | 0.02  (0.00, 0.04) | 1.16  (1.03, 1.31) | -0.02  (-0.04, 0.00) | 0.98  (0.86, 1.11) |
| *Rurality-Urbanicity* |  |  |  |  |
| Metropolitan | Ref-- | Ref-- | Ref-- | Ref-- |
| Micropolitan | 0.04  (0.03, 0.05) * | 1.15  (1.06, 1.24) * | -0.02  (-0.03, -0.01) * | 0.99  (0.91, 1.09) |
| Rural | 0.03  (0.00, 0.06) | 0.97  (0.81, 1.15) | -0.04  (-0.07, -0.01) * | 0.89  (0.73, 1.09) |
| *Dual-eligible* |  |  |  |  |
| Not Dual Eligible | Ref-- | Ref-- | Ref-- | Ref-- |
| Dual Eligible | 0.09  (0.08, 0.11) * | 1.43  (1.34, 1.53) * | 0.09  (0.08, 0.10) * | 1.25  (1.17, 1.35) * |
| *Number of Comorbidities* |  |  |  |  |
| 0 | Ref-- | Ref-- | Ref-- | Ref-- |
| 1 | 0.06  (0.05, 0.07) * | 1.22  (1.13, 1.31) * | 0.05  (0.04, 0.06) * | 1.18  (1.09, 1.28) * |
| 2 | 0.09  (0.08, 0.11) * | 1.44  (1.33, 1.56) * | 0.09  (0.07, 0.10) * | 1.33  (1.22, 1.45) * |
| 3+ | 0.15  (0.14, 0.16) * | 1.74  (1.62, 1.87) * | 0.18  (0.17, 0.19) * | 1.82  (1.69, 1.95) * |
| *Cancer Type* |  |  |  |  |
| Breast Cancer | Ref-- | Ref-- | Ref-- | Ref-- |
| Colorectal Cancer | 0.06  (0.04, 0.07) * | 1.24  (1.14, 1.34) * | 0.16  (0.14, 0.17) * | 1.35  (1.23, 1.47) * |
| Lung Cancer | 0.13  (0.12, 0.15) * | 1.54  (1.43, 1.66) * | 0.20  (0.19, 0.21) * | 1.83  (1.68, 2.00) * |
| *Cancer Stage* |  |  |  |  |
| 1 | Ref-- | Ref-- | Ref-- | Ref-- |
| 2 | 0.07  (0.06, 0.09) * | 1.23  (1.14, 1.34) * | 0.11  (0.10, 0.13) * | 1.29  (1.18, 1.40) * |
| 3 | 0.14  (0.12, 0.15) * | 1.40  (1.29, 1.51) * | 0.22  (0.20, 0.23) * | 1.50  (1.38, 1.63) * |
| 4 | 0.26  (0.25, 0.27) * | 2.05  (1.91, 2.21) * | 0.43  (0.42, 0.44) * | 2.47  (2.29, 2.67) * |
| *Year of Diagnosis* |  |  |  |  |
| 2018 | Ref-- | Ref-- | Ref-- | Ref-- |
| 2019 | -0.02  (-0.03, -0.01) * | 0.92  (0.88, 0.97) * | -0.01  (-0.02, -0.01) * | 0.97  (0.92, 1.02) |
| *Oncology Physician Count*  *Per Beneficiary in HRR* | -0.01  (-0.04, 0.02) | 1.01  (0.43, 1.03) | -0.02  (-0.04, 0.01) | 1.06  (0.51, 1.30) |
| *Medicare Advantage*  *Penetration Rate in*  *Patient's State of Residence* | -0.04  (-0.11, 0.03) | 0.67  (0.79, 1.08) | -0.04  (-0.12, 0.04) | 0.81  (0.86, 1.17) |

a. Probability of at least one visit vs. no visits.

b. Relative risk of additional visits conditional on at least one.

* Statistically significant at p-value <- 0.01.

SDI: social deprivation index.

**Supplemental Figure 4.** Proportion of the analytic cohort that had at least one emergency department visit and/or at least one non-elective hospitalization during the first 12 months after their initial cancer diagnosis, stratified by physician network vulnerability region.


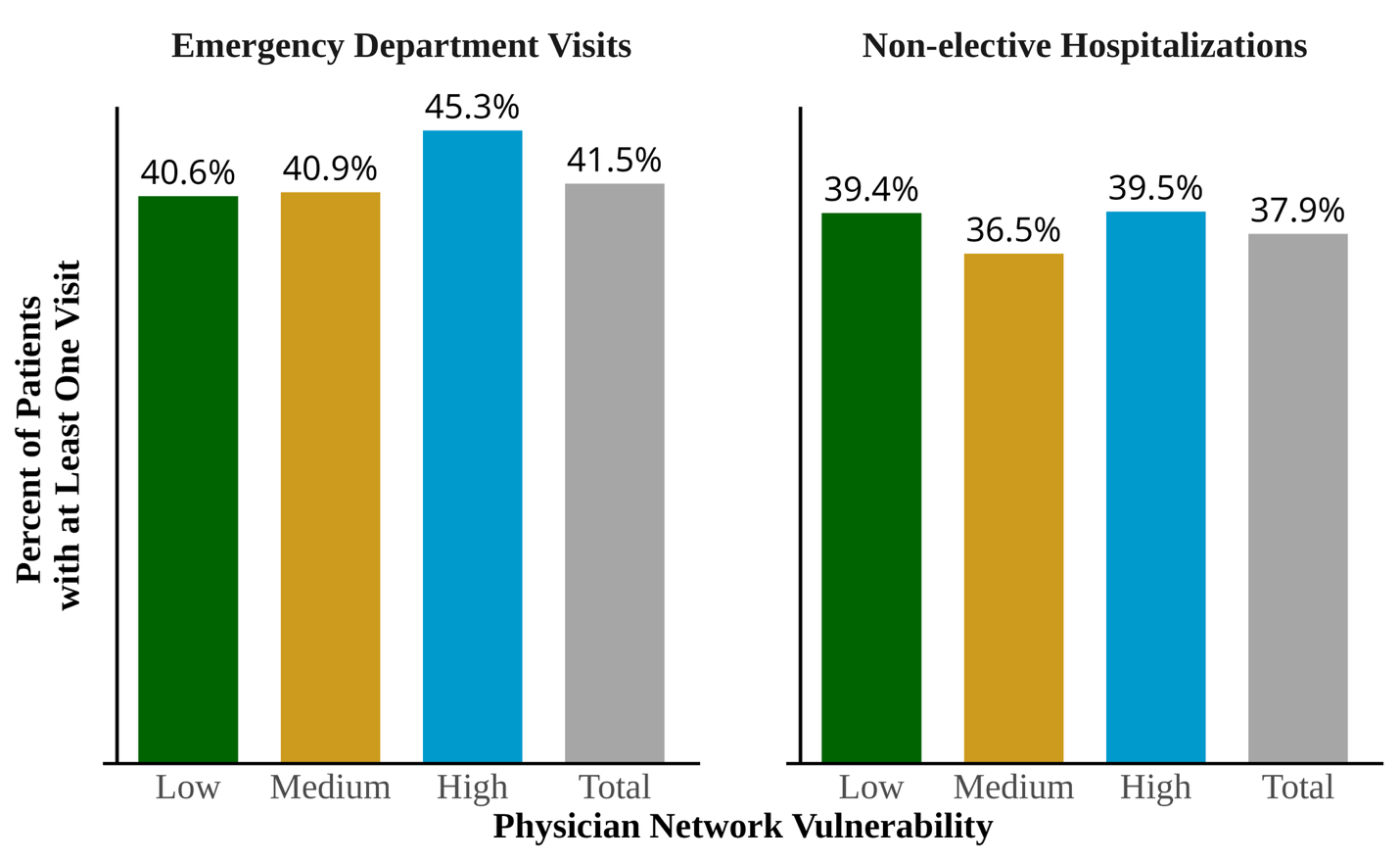


**Supplemental Figure 5.** Association between Social Deprivation Index (SDI) and Yost Index quintile among the analytic cohort.


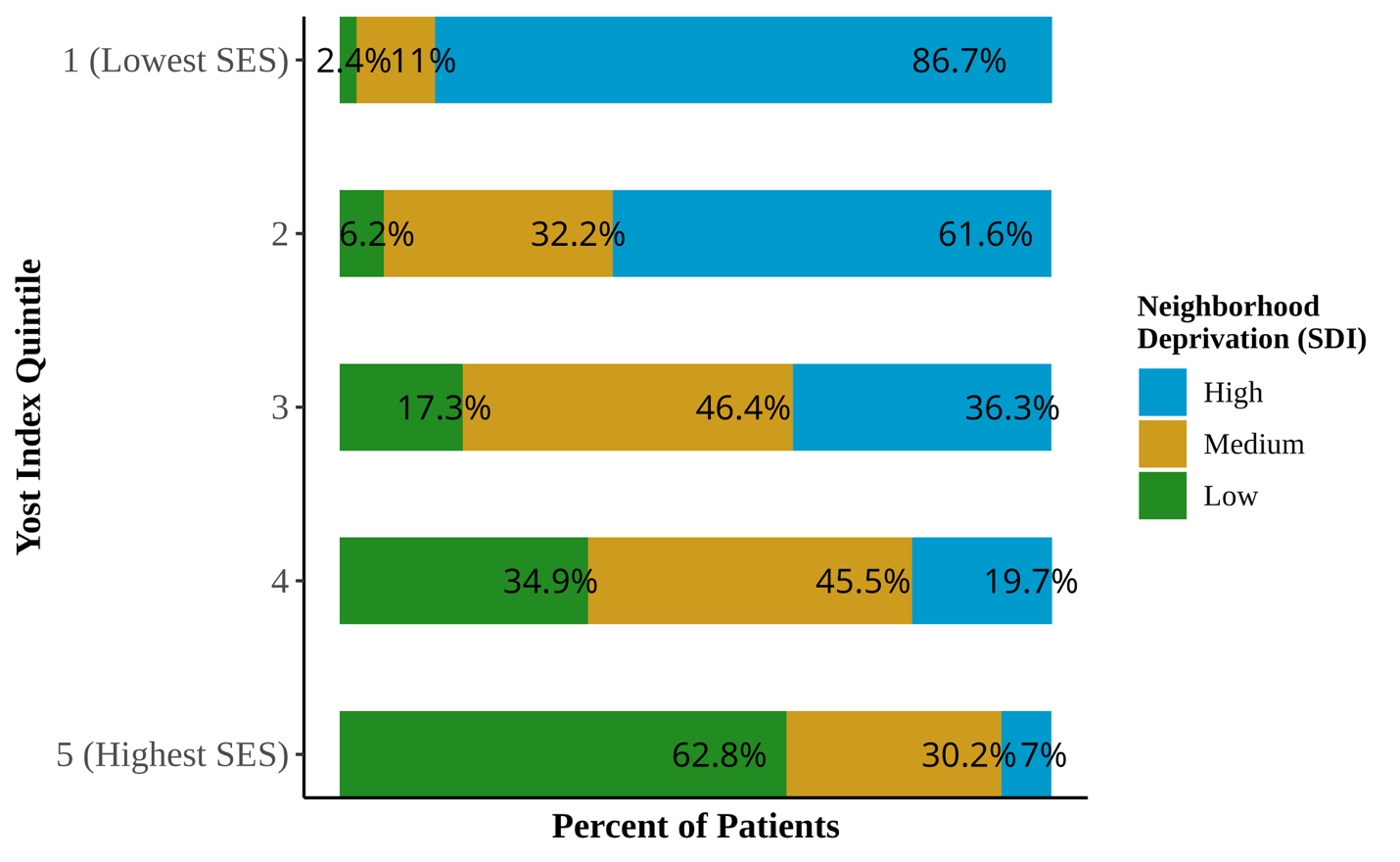


SDI: social deprivation index
